# Nurse practitioner medication abortion providers in Canada: results from a national survey

**DOI:** 10.1136/bmjsrh-2024-202379

**Published:** 2024-10-16

**Authors:** Emma Stirling-Cameron, Andrea Carson, Abdul-Fatawu Abdulai, Ruth Martin-Misener, Regina Renner, Madeleine Ennis, Wendy V Norman

**Affiliations:** 1 School of Population and Public Health, The University of British Columbia, Vancouver, British Columbia, Canada; 2 School of Nursing, Dalhousie University, Halifax, Nova Scotia, Canada; 3 School of Nursing, The University of British Columbia, Vancouver, British Columbia, Canada; 4 Department of Obstetrics and Gynaecology, The University of British Columbia, Vancouver, British Columbia, Canada; 5 Department of Family Practice, The University of British Columbia, Vancouver, British Columbia, Canada; 6 Faculty of Public Health and Policy, London School of Hygiene & Tropical Medicine, London, UK

**Keywords:** Health Services Research, surveys and questionnaires, Reproductive Health Services, Health Policy, family planning services, Abortion, Therapeutic

## Abstract

**Background:**

In 2017, nurse practitioners (NPs) became the first non-physician healthcare providers authorised to independently provide medication abortion (MA) in Canada. We aimed to report on demographic and clinical characteristics of NPs providing mifepristone/misoprostol MA in Canada and to identify context-specific barriers and enablers to NP provision of mifepristone/misoprostol MA in Canada among MA providers and non-providers.

**Methods:**

From August 2020 to February 2021, we invited Canadian NPs to complete a national, web-based, bilingual (English/French) survey. The survey was distributed through national and provincial nursing associations and national abortion health professional organisations. We collected demographic and clinical care characteristics and present descriptive statistics and bivariate analyses to compare the experiences of NP providers and non-providers of MA.

**Results:**

The 181 respondents represented all Canadian provinces and territories. Sixty-five NPs (36%) had provided MA at the time of the survey and 116 (64%) had not. Nearly half (47%) of respondents worked in rural or remote communities and 81% in primary care clinics. Significant barriers impacting non-providers’ abilities to provide MA included limited proximity to a pharmacy that dispensed mifepristone/misoprostol, few experienced abortion providers in their community of practice, poor access to procedural abortion services, policy restrictions in NPs’ places of employment, and no access to clinical mentorship. Some 98% of NPs providing MA services had never encountered anti-choice protest activity.

**Conclusions:**

NPs appear prepared and able to provide MA, yet barriers remain, particularly for NPs in smaller, lower-resourced communities. Our findings inform the development of supports for NPs in this new practice to improve abortion access in Canada.

**What is already known on this topic**

- Although in the UK and many countries only physicians may independently provide abortion services, Canada in 2017 authorized nurse practitioners (NPs) to independently prescribe mifepristone/misoprostol medication abortion (MA).

**What this study adds**

- Canadian NPs reported highly positive experiences to provide MA, particularly when they had working relationships with a local pharmacy team, with local abortion providers and/or with a clinical mentor. Barriers to the implementation of NP provided MA arose where there were long distances to procedural abortion services, and policy restrictions in NPs’ places of employment.

**How this study might affect research, practice, or policy**

- Abortion care provision by NPs is effective and acceptable and will contribute to improving abortion access. In light of current physician shortages, task shifting early medication abortion to NPs could contribute to improved access to primary care.

## Introduction

 In 2015, Health Canada approved the gold-standard first-trimester medication abortion (MA) drug combination, mifepristone (200 mg) and misoprostol (800 μcg; Mifegymiso), which became available in Canada for the first time in January 2017.^1^ Prior to the introduction of MA, abortion care in Canada was almost exclusively procedural (also known as surgical), was accessible through fewer than 100 clinics, predominately located in city centres, and only physicians were licensed to provide abortion care.^2^ Four years after mifepristone MA was approved in Canada, MA accounted for 39.5% of the 97 210 abortions reported in 2022,^3^ in stark contrast to under 4% pre-mifepristone.^4^ The ability to prescribe and dispense mifepristone allowed primary care practitioners across Canada to introduce MA into their scope of practice as an additional service available in their primary care practice.^5 6^ MA could be provided through any primary care clinic, no longer risking a need to cross protest lines outside abortion clinics, helping to destigmatise the experience of seeking abortion care, and to expand access points closer to patients’ homes.^7 8^ This new service has increased abortion access and offers pregnant people greater autonomy over their abortion experience.^9^

This shift in the Canadian abortion landscape was coupled with other major regulatory changes, notably, the November 2017 novel authorisation of nurse practitioners (NPs) to independently provide MA, where approved by their provincial licensing body, without physician oversight.^10 11^ This marked the first time in Canadian history when non-physician healthcare providers could independently provide abortion care.^8^ In Canada, there are over 6600 NPs. However, in general NPs work as employees in healthcare settings with a particular focus (eg, chronic disease specialty clinics, long-term care, community elder care) with an unknown but small proportion employed in sexual health or primary care settings among reproductive-aged people.^12 13^ They are healthcare professionals with additional graduate education and clinical practice experience,^14^ giving them the knowledge and skills to autonomously diagnose, order and interpret diagnostic tests, prescribe pharmacological treatment, and perform certain procedures.^13 14^ International literature has demonstrated that clinical outcomes among MAs provided by non-physician practitioners, including NPs, were equivalent to those provided by physicians.^15^,^17^

Expanding the NP scope of practice to include MA has the potential to increase access to abortion care, reduce geographical access disparities, and normalise abortion as a fundamental component of primary healthcare.^18 19^ While there is a critical need to understand the uptake of MA prescribing among NPs, there are little or no data on this new workforce and implementation of this new service. Our objectives were to (i) report on demographic and clinical characteristics of NPs providing mifepristone/misoprostol MA in Canada and (ii) identify context-specific barriers and enablers to NP provision of mifepristone/misoprostol MA in Canada.

## Methods

### Study design and setting

We conducted a mixed-methods, cross-sectional, national survey from August 2020 to February 2021. Findings from the qualitative arm of this project have been presented elsewhere.^8 20^ Any NP currently registered with a Canadian provincial/territorial college/regulatory body or Association of Nurses was eligible to participate in this study. Participants did not need to be a current medication provider to complete the survey. After data collection was completed, participants were divided into two groups: NPs who had previously provided MA in Canada, and those who had not (at the time of survey completion). We collected and managed study data using Research Electronic Data Capture (REDCap) tools hosted at the University of British Columbia.^21 22^

### Survey instrument

The survey was adapted from components of previous Contraception and Abortion Research Team (CART-GRAC) surveys focused on physician and pharmacist provision of MA in Canada.^23 24^ We designed and tested the survey iteratively with researchers (n=12) and clinicians experienced in the topic area (n=21) to ensure questions were appropriate for NP respondents. The survey question design utilised a framework based on an adaptation of the diffusion of innovations theory (Greenhalgh et al., 2004), which we used to understand how novel practices are adopted and implemented into health systems.^25^ In the context of implementing NP-prescribed MA, the diffusion of innovations theory can be used to identify key stakeholders, assess their willingness to adopt the innovation, and develop strategies to encourage adoption and implementation. The Greenhalgh framework consists of six overarching constructs, five of which were utilised in our study: (i) outer context; (ii) system antecedents; (iii) characteristics of the innovation; (iv) system readiness; and (v) characteristics of adopters and assimilators. The diffusion of innovations theory has been used in other quantitative and qualitative studies to examine the uptake of MA prescribing among physicians and pharmacists.^7 26 27^ We mapped areas of alignment of the specific domains and applicability to the novel practice for NP provision of MA to the diffusion of innovations framework to guide analysis (see table 1).

**Table 1:** Domains of the diffusion of innovations in health service delivery organisations, adapted from Greenhalgh et al. (2004)^25^ and applied to the novel implementation of nurse practitioner-provided medication abortion in Canada

Domain	Definition	Example MA provision factors
Outer Context	The outer context examines the sociopolitical climate, political directives, interorganisational settings and wider environmental factors that may affect an innovation.	Travel time to access management for uterine evacuation; Local attitudes toward abortion and anti-choice sentiments
System Antecedents for Innovation	System antecedents address the structural, cultural and organisational context for an innovation. This includes the potential context for change (eg, risk-taking climate, workplace structure), organisational structure and capacity for new knowledge.	Access to ultrasound; Time to obtain ultrasound; Access to a point-of-care ultrasound; Round trip travel time for patients to obtain ultrasound; Working in an interdisciplinary team; Impact of global pandemic
System Readiness for Innovation	System readiness refers to a system or organisation’s ability to prepare for the implementation of a specific innovation. This can include facets such as tension for change, innovation-system fit, implications of the innovations, support and advocacy, and time and resources.	Pharmacy nearby to dispense mifepristone/misoprostol; Communication with pharmacy about dispensation; Abortion services in community (procedural and medication); Abortion providers (NP, MD) in community; Workplace/employer policies allow for independent MA provision
Characteristics of the Intervention	The characteristics of an innovation refer to the complexity, trialability and availability of technical support to implement the innovation.	Estimates on requests for MA from patient population; Estimates on how many MAs providers have completed; Difficulty associated with providing MA
Adapters and Assimilators	This domain describes the characteristics of those who engaged in the innovation. This includes provider motivations and values, their social network, learning style/ability, access to training, needs and goals.	Abortion training in curriculum; Completed external training and type completed; Has or would like an abortion provider

MA, medication abortion; MD, doctor of medicine; NP, nurse practitioner.

The final survey instrument consisted of 99 items. We selected questions to capture NP demographics, details about practice settings and community, MA care processes (including use of virtual care), factors contributing to success, ongoing barriers to provision and access for patients. We offered the survey via a weblink with data collection directly into REDCap, with a choice of versions in English and French. The French version of the survey was professionally translated and reviewed by two native French speakers with experience in abortion care.

### Recruitment

As NP provision of abortion in Canada was a relatively new practice at the time of data collection, and there is no specific listing for NPs by their practice setting, we did not have any method to identify all NPs who were in an employment position suitable to potentially include the practice of MA. For example, many NPs work in geriatrics, complex chronic diseases, or maternal and newborn care practice settings where MA would not be offered. To reach nationally, and among as many eligible clinicians as possible, we distributed a generic, bilingual survey link through multiple collaborating healthcare professional organisations and networks (eg, British Columbia Nurse Practitioners Association; L'Association des Infirmières Praticiennes Spécialisées du Québec (AIPSQ); Nurse Practitioners Association of Canada; the National Abortion Federation (NAF) Canada and through our web-based community of abortion practice (www.caps-cpca.ubc.ca)) inviting NPs to participate in our study. We requested that associations distribute survey links through existing mailing lists and social media. Associations were asked to send a minimum of two survey invitations over the course of the study period. All participants were entered into a draw for the chance to win an iPad and one participant was selected at the end of data collection.

### Statistical analyses

We conducted descriptive analyses and bivariate comparisons using independent sample *t*-tests and χ-square analyses using SPSS 28. We included questions that were not answered by all study respondents. The denominator used in each reported percentage is the number of respondents who answered that individual question.

### Ethics approval

The project was approved by the Research Ethics Boards at Dalhousie University and the University of British Columbia.

## Results

We present the findings of this research study in two sections, (i) participant characteristics (table 2) and (ii) factors related to readiness for diffusion of this innovation (ie, novel NP provision of MA in Canada, tables3  4).

**Table 2:** Demographic characteristics of nurse practitioner providers and non-providers of medication abortion across Canada (N=181)

Demographic characteristic	Providersn (%)	Non-providersn (%)	Totaln (%)
Age (years)			
25–34	9 (15)	26 (24)	35 (21)
35–44	24 (39)	43 (39)	67 (39)
45–54	17 (28)	28 (26)	45 (26)
55–65	10 (16)	11 (10)	21 (12)
66+	<5	<5	*
Province			
Prairies (AB+SK+ MB)	6 (9)	39 (34)	45 (25)
BC	10 (15)	20 (17)	30 (17)
ON	35 (54)	13 (11)	48 (27)
Quebec	0 (0)	17 (15)	17 (9)
Atlantic Canada (NS+NB+PEI+NL)	12 (18)	23 (20)	35 (19)
Northern (NT+YK+NU)	<5	<5	*
Setting			
Rural	21 (42)	34 (50)	55 (47)
Urban	29 (58)	34 (50)	63 (53)
Employer			
Provincial	56 (86)	94 (81)	150 (83)
Federal	0 (0)	<5	*
Private	<5	9 (8)	*
Others	6 (9)	9 (8)	15 (8)
Specialty			
Reproductive health/sexual health/women’s health	8 (12)	10 (9)	18 (10)
Primary care/family practice	55 (85)	91 (78)	146 (81)
Other	<5	15 (13)	*
Gender			
Woman	61 (94)	106 (91)	167 (92)
Man	3 (4)	9 (8)	12 (7)
Other	<5	<5	*

* Cell counts below five not reported to maintain participant anonymity.

AB, Alberta; BC, British Columbia; MB, Manitoba; NB, New Brunswick; NL, Newfoundland and Labrador; NS, Nova Scotia; NT, Northwest Territories; NU, Nunavut; ON, Ontario; PEI, Prince Edward Island; SK, Saskatchewan; YK, Yukon.

**Table 3:** Survey data responses stratified by nurse practitioner medication abortion providers and non-providers and categorised by diffusion of innovations domains (N=181)

Practice Factor	NP providers n (% within providers) (N=65)	NP non-providers n (% within non-providers) (N=116)	P value
**Outer contextual factors**			
Procedural management option for failed MA is available locally (eg, suction evacuation)	40 (64.5)	61 (59.2)	0.515
Travel time to uterine haemorrhage management			0.578
Less than 2 hours	56 (91.8)	90 (89.1)	
More than 2 hours	5 (8.2)	11 (10.9)	
Procedural management for incomplete miscarriage is available	47 (77.0)	75 (72.8)	0.507
Anti-choice protest activity experienced	*	–	–
Anticipation or uncertainty around anti-choice protest activity	–	48 (47.0)	–
**System antecedents**			
Access to ultrasound in the community	57 (91.9)	31 (88.6)	0.583
Access to a point-of-care ultrasound	9 (14.5)	5 (14.3)	0.975
Round trip travel time for patients to get ultrasound			0.058
Less than 2 hours	49 (92.4)	30 (100.0)	
More than 2 hours	6 (5.7)	0 (0.0)	
**System readiness for innovation**			
Pharmacy nearby to dispense mifepristone/misoprostol†	53 (85.5)	29 (28.2)	<0.001
Communication with pharmacy about dispensation†	49 (79.0)	6 (19.4)	<0.001
Abortion services in community			
Procedural abortion†	15 (24.6)	7 (6.9)	<0.001
MA	11 (18.0)	20 (19.6)	0.97
Both	22 (36.1)	55 (53.9)	0.077
Neither/I don't know	17 (27.9)	23 (22.5)	0.325
Abortion providers in community			
NPs†	54 (87.1)	30 (29.4)	<0.001
Physicians†	47 (75.8)	65 (63.7)	0.031
Neither/I don’t know†	*	34 (33.3)	<0.001
**Adopters andassimilators**			
Abortion training in NP curriculum	*	*	0.578
Completed external training†	57 (93.0)	21 (21.0)	<0.001
Training type completed‡			
SOGC	52 (93.0)	11 (55.0)	–
Manufacturer	0 (0.0)	*	–
CAPS/NAF workshop	*	*	–
Colleague/mentor/clinic	*	5 (25.0)	–
Has an abortion mentor	33 (54.0)	16 (47.0)	0.510
Desires an abortion mentor†	29 (48.0)	62 (64.0)	<0.001

* Cell counts below five not reported to maintain participant anonymity.

† Indicates statistically significant value (*p*p<0.05).

‡ Respondents could select more than 1one answer option.

§ Unless indicated otherwise. Percentages were calculated based on the total number of respondents for the individual variable (based on skip-pattern logic and nonmandatory questions). The denominator for each reported percentage consists of the number of respondents who answered that question.

CAPS, Canadian Abortion Providers Support; MA, medication abortion; NAF, National Abortion Federation Canada; NP, nurse practitioner; SOGC, Society of Obstetricians and Gynaecologists of Canada .

**Table 4:** Barriers and potential facilitators disaggregated by nurse practitioner provider status (N=181)

Practice Factor	MA provider (N=65)	MA non-provider (N=116)	95% CI
	Mean (SD)	Mean (SD)	
Barriers*			
Skill required	3.4 (0.9)	3.1 (1.0)	−0.01; 0.61
Training	3.2 (0.7)	2.9 (0.9)	−0.02; 0.58
Logistics	2.8 (0.9)	2.5 (0.9)	−0.06; −0.55†
Access to a clinical protocol	3.4 (0.9)	2.8 (1.1)	0.28; 0.95†
Time required	3.0 (0.9)	2.9 (1.0)	−0.22; 0.46
Policy restrictions in place of employment	3.8 (1.0)	2.8 (1.0)	0.66; 1.50†
Potential facilitators‡			
Access to timely ultrasound	2.7 (1.7)	3.6 (1.6)	−1.40; −0.36†
Mentorship from another NP MA provider	2.2 (1.4)	4.0 (1.5)	−2.25; −1.35†
Mentorship from a physician MA provider	2.4 (1.5)	4.0 (1.4)	−2.12; −1.18†
Access to procedural abortion for failed MA	2.7 (1.5)	2.9 (1.7)	−0.72; 0.33
Pharmacy willing to dispense	2.3 (1.6)	3.0 (1.7)	−1.30; −0.23†

Responses were recorded on a five-point Likert scale: Strongly disagree/not a barrier=1, Strongly agree/would facilitate access=5.

* Barrier question phrased as, “Difficulty of providing MA: “Extremely difficult=1; Very easy=5”.

† Indicates statistically significant value (at pp<0.05).

‡ Facilitator questions asked, “Tthe following would make it easier for me to provide medication abortion: if I had access to…”.

CI, confidence interval; MA, medication abortion; NP, nurse practitioner; SD, standard deviation.

### Participant characteristics

A total of 181 NPs completed the survey. Demographic data for all NP respondents are listed in table 2, stratified by provision experience (ie, provider vs non-provider) at the time of survey completion. NPs from every Canadian province and territory participated, with 47% (n=55) from rural communities.^28^ Sixty-five NPs (36%) had provided at least one MA at the time of the survey. Among NPs not currently providing MA, 21.5% (n=39) indicated that they intend to provide in future, 27.1% (n=49) were unsure and 15.5% (n=28) did not intend to provide MA.

### Readiness for diffusion of NP-provided MA

We report here on the factors related to the diffusion of the innovation, with “innovation” referring to the novel NP provision of MA in Canada. Factors include those related to (i) the outer context, (ii) system antecedents, (iii) characteristics of the innovation, (iv) system readiness for the innovation and (v) adopters and assimilators.

### Outer contextual factors to abortion provision

No statistically significant differences were observed between NP MA providers and non-providers with regard to outer contextual factors (table 3). Fewer than five participants (<2%) among NP providers indicated they had encountered any anti-choice protest activity. However, among NPs who had not yet provided MA, 47% (n=48) indicated apprehension around encountering anti-choice activity.

### System antecedents

No statistically significant differences were observed between NP MA providers and non-providers within system antecedents (table 3). Among NP respondents who had previously provided MA, 58% (n=36) worked in interdisciplinary teams among whom others also provided MA, 92% (n=57) had access to ultrasound in their community of practice and 85% (n=53) reported that their patients could access ultrasound for gestational dating within 7 days. Seventy-three percent (n=47) of NP MA providers worked in community care clinics with 17% (n=11) working in NP-led clinics or emergency or hospital-affiliated service centres. All NP providers were compensated for their employment via salaried pay.

Provision of virtual or telemedicine MA prior to 2020 more than doubled to provision by more than two-thirds of NP MA provider respondents during the COVID-19 pandemic. Twenty-nine percent (n=18) of NPs were offering virtual abortion care in some capacity before the COVID-19 pandemic and 69% (n=43) provided virtual abortion care during the COVID-19 pandemic.

### Characteristics of the innovation

On average, responding NP MA providers completed 20 MAs per year (M=20, SD=40), with a range from 0 to 200 per provider in the past year (0 indicating that some NPs had previously provided MA, but had not in the past 12 months), although most had provided 15 or fewer abortions in the past year (2019/2020). Most followed package-label indications providing MA up to 9 weeks’ gestation, while a quarter of providers followed the Society of Obstetricians and Gynaecologists of Canada (SOGC) national clinical practice guidelines to provide MA up to 10 weeks’ gestation.

### System readiness for innovation

Statistically significant differences were observed between MA providers and non-providers related to system readiness (see table 3). Providers were significantly more likely to report having an NP or physician abortion provider working in their community when compared with non-providers. They were also significantly more likely to have procedural abortion services located in their community, although the rate was low among both providers and non-providers. NP providers were significantly more likely to have ongoing communication with a local pharmacy about dispensation of MA when compared with non-providers.

Perceived barriers and potential facilitators to providing MA are provided in table 4. The logistics of providing MA, access to a clinical protocol, and policy restrictions in NPs’ places of employment were significantly different between NP MA providers and non-providers, with non-providers reporting greater levels of perceived difficulty compared with providers. Access to ultrasound, a NP or physician mentor, and a pharmacy willing to dispense mifepristone were significantly more likely to be reported as potential enablers for non-providers compared with providers.

### Adopters and assimilators

Statistically significant differences were observed between MA providers and non-providers related to clinical preparation for provision of a new service (see table 3). Providers were significantly more likely to have completed external training compared with non-providers, with training offered by the SOGC reported most often across groups. Both groups of NPs indicated a high rate of desire for a mentor who could advise on MA, though non-providers reported this significantly more often. Abortion training delivered as part of NP curriculum was low across both providers and non-providers.

## Discussion

For the first time in Canadian history, NPs can independently prescribe first-trimester MA. To our knowledge, this is the first quantitative exploratory study of the novel innovation of NP-provided MA in Canada. We found that NPs were facilitated to provide MA when they had access to and positive communication with a local pharmacy team and/or with other NP or physician abortion providers in their community of practice. NP MA providers almost never experienced anti-choice protest activity. However, our findings indicated that several key diffusion of innovations^25^ constructs impeded NPs’ abilities to offer MA to their patients, including access to a clinical mentor, proximity to a pharmacy willing to dispense mifepristone/misoprostol, access to procedural abortion services, and policy restrictions in NPs’ places of employment.

Our study found that the provision of MA was more easily facilitated when NPs were working in communities where other abortion providers were present and when they had access to clinical mentorship from physicians or NPs. This corroborates findings from recent qualitative research in this area^8 20^ which documented the support and reassurance mentors offered to new MA providers as they encountered novel presentations or complications while guiding their own patients through MA. Our results further detailed a significant association between MA provider status and the presence of other NP abortion providers in the community. This and other previous Canadian research indicate that NPs are an essential source of leadership with regard to onboarding new NPs into MA care and are serving to facilitate the education and promotion of abortion care engagement among healthcare workers.^8^

Our analysis further found that even NPs with experience in providing MA still expressed a wish for the support of a mentor. Abortion mentorship networks (eg, the Australian Contraception and Abortion Primary Care Practitioner Support (AusCAPPS) Network, Canadian Abortion Providers Support (CAPS) Network) have been established nationally and internationally with the intent to support the increased availability and success of abortion providers.^29 30^ Such networks facilitate the exchange of best practice advice, assist in the location of pharmacies that dispense mifepristone, and enable clinicians to remain abreast of shifting policy and regulatory changes and guidelines. The continued expansion of geographical or profession-specific networks may help to improve health professional knowledge, attitudes and provision of MA, and other aspects of reproductive care (eg, long-acting contraceptives).^30^

Our study also found low rates of abortion-related curriculum in NP education programmes across both groups of providers and non-providers. It is important to note that many participants in this study completed their MA training prior to the implementation of mifepristone in Canada. However, companion qualitative work to our study noted gaps in nursing education around abortion care, with NPs seeking out training from mentors or professional bodies (eg, SOGC), further demonstrating the critical role that these abortion champions play.^8 18 19^ Important efforts are being made in this area. For example, an interprofessional health education course has been formed at Dalhousie University in Halifax, Canada to educate health professional trainees, including nursing students, on the legal history of abortion in Canada, clinical guidelines for medication and aspiration abortion, socio-emotional and therapeutic support, and information on contraception.^31^ Further research is needed to explore what, if any, curriculum is being delivered through NP programmes and how that may be better supported.

Interprofessional collaborations with laboratory, ultrasound and pharmacy services were reported as key factors for the success among MA providers in this study. Communication with local pharmacies that regularly stocked and dispensed mifepristone facilitated NPs ability to provide MA. Other Canadian literature has found that positive communication between MA providers and pharmacists helped to maintain mifepristone stock in pharmacies and allowed patients to be cohesively supported by both practitioners throughout the abortion process.^26 27 32^ Integral to these relationships was a mutual belief that abortion is ethical, safe and beneficial to the community.^8^

We found that more than 98% of NP MA providers reported never having experienced anti-choice protest activity, yet apprehension related to potential anti-choice protesting was reported by almost half of NP MA non-provider participants in our survey. This is consistent with other Canadian literature that shows that actual anti-choice violence or rhetoric targeted at clinics and providers remains low.^33 34^ Moreover, the expansion of MA into primary care reduces the targetability of individual clinics and hospitals and helped to normalise abortion care as an aspect of everyday healthcare.^8 20^

### Strengths and limitations

The main limitation of this study was its exploratory nature and its convenience sampling strategy. The survey was distributed during the first two waves of the COVID-19 pandemic when many care providers were inundated with increased clinical responsibilities. To mitigate this, we made repeated recruitment invitations over 7 months, consistent with other abortion healthcare professional surveys led by our team during the pandemic^5 35^ and expanded the number of organisations we included in our initial recruitment plan. Moreover, the exact number of NPs that practise in a setting that might potentially be appropriate for provision of MA in Canada remains unknown. Though the Canadian Institute for Health Information tracks the number of practising NPs (6600 in 2022), the proportion who have provided MA since it was included the scope of practice for NPs (2017) is not known. Therefore, we were unable to estimate a response rate for the survey. The main strength of our data is the national distribution of our sample of respondents and in the nearly equal distribution of rural and urban practitioners, obtaining information on a wide array of practice setting and provincial/territorial contexts. To our knowledge, this is the only quantitative study documenting the barriers and facilitators to the novel implementation of NP-provided MA in Canada.

## Conclusions

Our findings add to the growing body of international literature that demonstrates that NPs are well-suited to providing MA, but further support is needed to address remaining barriers to implementation. The success of NPs in providing abortion care is crucial for reducing barriers to patient access and may help to alleviate inequitable or delayed access to abortion care because of the national physician shortage and the vast rural geography of Canada.

## Data Availability

No data are available.
